# High-throughput *in situ* experimental phasing

**DOI:** 10.1107/S2059798320009109

**Published:** 2020-07-28

**Authors:** Joshua M. Lawrence, Julien Orlans, Gwyndaf Evans, Allen M. Orville, James Foadi, Pierre Aller

**Affiliations:** a Diamond Light Source, Harwell Science and Innovation Campus, Didcot OX11 0DE, United Kingdom; b UMR0203, Biologie Fonctionnelle, Insectes et Interactions (BF2i); Institut National des Sciences Appliquées de Lyon (INSA Lyon); Institut National de Recherche pour l’Agriculture, l’Alimentation et l’Environnement (INRAE), University of Lyon (Univ Lyon), F-69621 Villeurbanne, France; c Research Complex at Harwell, Rutherford Appleton Laboratory, Didcot OX11 0FA, United Kingdom

**Keywords:** *in situ* crystallography, experimental phasing, crystal soaking, high throughput, isomorphism

## Abstract

In this article, a new approach to experimental phasing for macromolecular crystallography (MX) at synchrotrons is introduced and described for the first time. It makes use of automated robotics applied to a multi-crystal framework in which human intervention is reduced to a minimum. Hundreds of samples are automatically soaked in heavy-atom solutions, using a Labcyte Inc. Echo 550 Liquid Handler, in a highly controlled and optimized fashion in order to generate derivatized and isomorphous crystals. Partial data sets obtained on MX beamlines using an *in situ* setup for data collection are processed with the aim of producing good-quality anomalous signal leading to successful experimental phasing.

## Abstract 2

A new procedure for experimental phasing at room temperature using diffraction data collected directly from the crystallization plates is described, demonstrated and tested.

## 1. Introduction   

The exploitation of multiple crystals is becoming common in data collection and analysis at synchrotrons and X-ray free-electron lasers (XFELs); the encompassing methods are often termed serial macromolecular crystallography (SMX). Indeed, new scenarios have first been envisaged and then implemented to increase the probability of obtaining complete and good-quality sets of reflections by combining data from several to thousands of crystals in creative ways. These strategies have been applied in merging partial data sets from membrane proteins and large complexes (Arakawa *et al.*, 2015 ▸; Axford *et al.*, 2012 ▸, 2015 ▸; Huang *et al.*, 2015 ▸, 2016 ▸; Mylona *et al.*, 2017 ▸), in methodologies adopted to reduce radiation-induced global damage (Garman, 2010 ▸; Garman & Owen, 2006 ▸; Owen *et al.*, 2006 ▸, 2014 ▸) and in the enhancement of anomalous signal for SAD phasing (Giordano *et al.*, 2012 ▸; Liu *et al.*, 2011 ▸, 2012 ▸, 2013 ▸; Rose *et al.*, 2015 ▸; Terwilliger *et al.*, 2016 ▸). It has been particularly enlightening and encouraging to discover that the impact of radiation-induced alterations is minimized by distributing the dose required to solve high-resolution structures over many samples, even without the need for crystal cryocooling. While a clear advantage of working at 100 K versus room temperature (RT) is to slow the global radiation-induced alterations in the sample, flash-cooling often introduces stress and deformations into the crystals. If, on the one hand, a higher X-ray dose can be applied to crystals at 100 K, thus making it possible to collect complete data sets, then, on the other hand, serious non-isomorphism between samples can limit their utility for phasing with isomorphous derivatives. Typically, RT crystallography is used in the early stage of the crystallization process to screen potential hits *in situ* directly in the crystallization plate (Aller *et al.*, 2015 ▸; Axford *et al.*, 2012 ▸; Bingel-Erlenmeyer *et al.*, 2011 ▸; Douangamath *et al.*, 2013 ▸; le Maire *et al.*, 2011 ▸; Sanchez-Weatherby *et al.*, 2019 ▸). However, the technique has been successful in facilitating high-throughput ligand-binding studies (Gelin *et al.*, 2015 ▸), in solving viral structures (Axford *et al.*, 2012 ▸) and those of membrane proteins (Axford *et al.*, 2015 ▸), and in providing more physiologically relevant RT structures of immuno­globulins (Davies *et al.*, 2017 ▸). In the present work, we demonstrate that the benefits of RT crystallography can be extended to experimental phasing by exploiting tens to hundreds of isomorphous crystals.

Experimental phasing techniques make use of heavy-atom (HA) derivatives, often in the form of halides, heavy metals or seleniomethionyl (SeMet) protein crystals, alongside native data, to allow *de novo* phasing of structures. The technique is most effective if the various crystals involved are all isomorphous to each other (Crick & Magdoff, 1956 ▸; Rould, 2007 ▸). RT crystals do not experience the potential alterations induced by cryocooling and are therefore, in principle, better suited for experimental phasing. However, owing to limitations on the total X-ray dose, RT methods most often yield incomplete wedges of data from each crystal; thus, it is necessary to merge partial data sets from several crystals into complete data sets. The need to use many different crystals also introduces the significant possibility of non-isomorphism. To overcome this limitation, the *BLEND* program (Foadi *et al.*, 2013 ▸) can select several groups of isomorphous crystals and then scale and merge the related intensities into a single, complete reflection file. Data sets are grouped with hierarchical cluster analysis, using unit-cell parameters as a critical descriptor.

**Figure 1:**
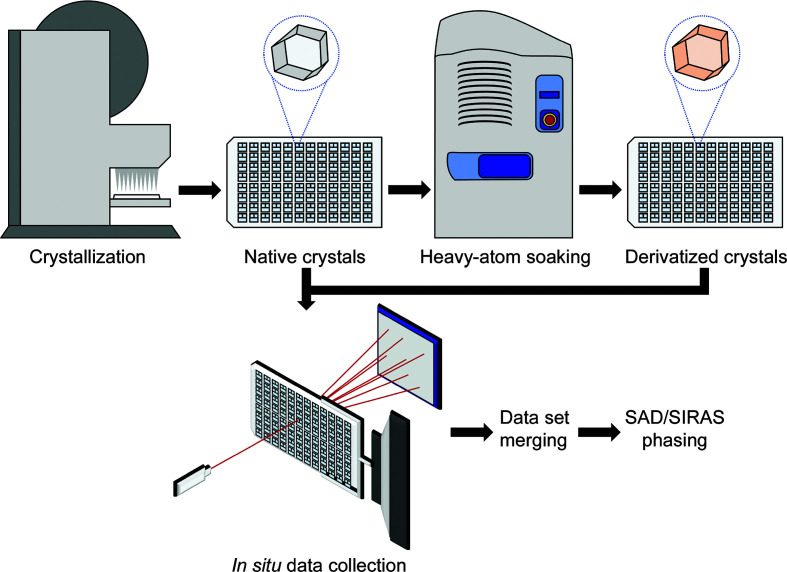
High-throughput workflow from crystallization to phasing. A video showing the workflow is available at https://www.diamond.ac.uk/Instruments/Mx/XFEL-Hub/Staff/Aller/Research.html.

It is, of course, of the utmost importance to have tens to hundreds of isomorphous crystals at hand for SMX methods. Farley *et al.* (2014 ▸) have convincingly argued that the lack of reproducibility of unit-cell parameters is mostly caused by the manual handling associated with crystal preparation and HA soaks. Furthermore, micropipettes dispensing sub-microlitre volumes of HA solution imply a certain degree of experimental error which changes the concentration of HAs, thus potentially causing the distortion of unit-cell parameters during the process (Garman & Murray, 2003 ▸; Pike *et al.*, 2016 ▸; Lu & Sun, 2014 ▸). In this study, we have developed a high-throughput, robot-assisted workflow using a robotic liquid dispenser (TTP Labtech Mosquito Crystal) and an acoustic droplet ejector (Labcyte Inc. Echo 550 Liquid Handler) for the production of HA-derivatized isocrystals for *in situ* data collection (Newman *et al.*, 2005 ▸; Villaseñor *et al.*, 2012 ▸; Chilingaryan *et al.*, 2012 ▸; Fig. 1 ▸). This workflow has been applied to test crystals of lysozyme and proteinase K soaked in four different halides and four different heavy-metal solutions. A comparison with control data sets from crystals grown and derivatized using the same method but cryocooled for data collection at 100 K offers an effective way to determine the penalty of the lack of isomorphism caused by manual handling and cryocooling of samples. The overall workflow allows the *de novo* solution of protein crystal structures using SAD and SIRAS phasing on a shorter timescale than that for standard cryocooling experiments (Pape & Schneider, 2004 ▸; Sheldrick, 2010 ▸; Taylor, 2010 ▸). This procedure can be achieved at the new VMXi beamline at Diamond Light Source (DLS), which is dedicated to *in situ* measurements (Sanchez-Weatherby *et al.*, 2019 ▸).

## 2. Materials and methods   

### 2.1. Sample preparation   

In order to ensure reproducibility and reduce human error, all sample crystallizations were handled using a TTP Labtech Mosquito Crystal with low-background 96-well Greiner CrystalQuick X crystallization plates optimized for *in situ* data collection (Aller *et al.*, 2015 ▸; Axford *et al.*, 2012 ▸, 2016 ▸).

#### 2.1.1. Lysozyme crystallization   

Commercial lyophilized lysozyme from hen egg white (Sigma–Aldrich) was resuspended in Milli-Q water to a concentration of 100 mg ml^−1^. Sitting drops were dispensed with the TTP Labtech Mosquito Crystal by mixing 100 nl lysozyme solution and 100 nl reservoir solution (1.0 *M* NaCl, 50 m*M* sodium acetate pH 4.5), and were equilibrated against 40 µl reservoir solution at 20°C. Crystals (200 × 200 × 50 µm) grew overnight and were found to belong to space group *P*4_3_2_1_2, with unit-cell parameters *a* = *b* ≃ 79, *c* ≃ 38 Å.

#### 2.1.2. Proteinase K crystallization   

Commercial lyophilized proteinase K (Sigma–Aldrich) was resuspended at 15 mg ml^−1^ in 50 m*M* HEPES pH 7.0. Sitting drops were dispensed with the TTP Labtech Mosquito Crystal by mixing 100 nl protein­ase K solution and 100 nl reservoir solution (1.2 *M* ammonium sulfate, 0.1 *M* Tris–HCl pH 8.0), and were equilibrated against 40 µl reservoir solution at 20°C. Microcrystals (10 × 10 × 10 µm) grew overnight and were found to belong to space group *P*4_3_2_1_2, with unit-cell parameters *a* = *b* ≃ 68, *c* ≃ 103 Å.

### 2.2. Crystal soaking   

Two crystallization wells per reservoir containing crystals were automatically soaked with a variety of halide and heavy-metal solutions using a Labcyte Inc. Echo 550 Liquid Handler in a similar way to its use by the XChem facility at DLS to inject structural fragments (Collins *et al.*, 2017 ▸). The liquid handler does not use pipette tips to transfer the soaking solution but uses a piezo-electric transducer, which generates a focused acoustic wave on the meniscus of the soaking solution to produce a 2.5 nl droplet (Hadimioglu *et al.*, 2016 ▸) directed on the crystal-containing drop. The Echo liquid handler allows highly reproducible and accurate crystal soaking for a high-throughput platform.

The halide solutions used were sodium bromide (NaBr), potassium bromide (KBr), sodium iodide (NaI) and potassium iodide (KI), all of which were purchased from Sigma–Aldrich. Stocks of 2 *M* halide solutions were prepared by dissolving the halide in the crystallization mother liquor. Halides tend to bind to patches on the surface of proteins through ionic interactions rather than through specific interactions with residue side chains (Pike *et al.*, 2016 ▸; Lu & Sun, 2014 ▸).

The heavy-metal solutions used were potassium tetranitroplatinate(II) [K_2_Pt(NO_2_)_4_], potassium tetrachloroaurate (KAuCl_4_·H_2_O), potassium hexachloroiridate (K_2_IrCl_6_) and samarium(III) chloride hexahydrate (SmCl_3_·6H_2_O), all of which were purchased from Hampton Research. Stocks of heavy-metal solutions at 100 m*M* were prepared by dissolving the heavy-metal compounds in the crystallization mother liquor. This combination of HAs were chosen based on their high chance of success (HA binding), their binding sites and their nontoxicity (Agniswamy *et al.*, 2008 ▸; Joyce *et al.*, 2010 ▸).

The final soaking concentrations used ranged from a few millimolar for heavy-metal solutions to 50–180 m*M* for halide solutions. To minimize crystal damage and retain crystal isomorphism, we performed RT short soaks of approximately 15 min for heavy metals and approximately 30 min for halides.

Lysozyme crystals were soaked in halide and heavy-metal solutions with final concentrations of 180 and 9 m*M*, respectively. KAuCl_4_·H_2_O was used at 4.5 m*M* because at 9 m*M* the lysozyme crystals start dissolving, likely owing to disruption of the crystal lattice on binding gold. For *in situ* data collection the soaking time varied between the first and the last data set collected. For cryogenic data collection, the soaking time was about 15 min for heavy-metal soaks and 30 min for halide soaks prior to rapidly plunge-cooling the samples in liquid N_2_.

Proteinase K crystals were soaked in halide and heavy-metal solutions with final concentrations of 50 and 2.5 m*M*, respectively. Half of the concentration was again used for KAuCl_4_·H_2_O, as a solution of KAuCl_4_·H_2_O at 2.5 m*M* was detrimental to proteinase K crystals. For *in situ* data collection the soaking time varied between the first and the last data set collected. For cryogenic data collection, the soaking time was about 15 min for heavy-metal soaks and 30 min for halide soaks.

The details of the soaking times and concentrations are shown in Table 1 ▸ for lysozyme and in Table 2 ▸ for proteinase K. Short soaks at a higher HA concentration were chosen based on the suggestion that they increase the chance of HA binding and yield more isomorphous crystals (Sun *et al.*, 2002 ▸). Short soaks also allowed for rapid data collection, ensuring the high-throughput nature of the workflow. Owing to the logistics of RT data collection, soak times for *in situ* samples tended to be slightly longer than for cryocooled samples as the crystals remain soaked in the HA throughout data collection.

We used a lower concentration for the heavy-metal conditions (less than 10 m*M*), as advised in the literature (Garman & Murray, 2003 ▸; Pike *et al.*, 2016 ▸). Also, heavy metals absorb large doses of X-rays owing to their greater number of excitable electrons, meaning that high concentrations would impact upon data quality owing to radiation damage (Pike *et al.*, 2016 ▸). For proteinase K, a quarter of the volume of each HA solution was used owing to the smaller size of the crystals.

### 2.3. Data collection   

#### 2.3.1. Cryo-data collection   

A subset of the crystals (natives and derivatives) were cryocooled in order to probe crystal isomorphism and HA binding at 100 K.

*Lysozyme*. The crystals were cryoprotected by adding ethylene glycol to 25%(*v*/*v*) to the crystal-containing droplets before flash-cooling in liquid nitrogen. For the derivatives, a heavy-metal/halide solution was added using the Echo liquid handler before adding the ethylene glycol solution prior to flash-cooling. At least five 360° rotation ‘complete’ data sets were collected from different crystals for the various native and derivative crystals, as shown in Supplementary Table S1. Data sets were collected on the I04 beamline at DLS using a beam size of 32 × 20 µm and a flux of ∼1.7–2.5 × 10^11^ photons per second.

*Proteinase K*. The crystals did not require cryoprotection before flash-cooling in liquid nitrogen. A slurry of microcrystals was mounted on a micromesh (10 µm aperture) from MiTeGen after adding heavy-metal/halide solution with the Echo liquid handler for the derivatized crystals. About 20 data sets with 50° rotation were collected for each condition (native and derivatives; Supplementary Table S2). Data collection was performed on the I24 beamline at DLS using a beam size of 9 × 6 µm and a flux of ∼2–4 × 10^11^ photons per second.

#### 2.3.2. *In situ* data collection   

All *in situ* data sets were collected on the I24 beamline at DLS (Aller *et al.*, 2015 ▸; Axford *et al.*, 2012 ▸, 2015 ▸). The CrystalQuick X plates were mounted manually on the horizontal goniometer.

*Lysozyme*. About 25 ‘incomplete’ data sets of 20° rotation were collected for each condition (Supplementary Table S3) using a beam size of 50 × 50 µm and a flux of about 2 × 10^11^ photons per second.

*Proteinase K*. About 50 ‘incomplete’ data sets of 2.5–5° rotation were collected for each condition (Supplementary Table S4) using a beam size of 9 × 6 µm and a flux of about 2 × 10^11^ photons per second.

The energy used varied depending on the HA condition for the crystals (Supplementary Table S1). Native data were collected at 12 660 eV on beamline I04 and at 12 800 eV on beamline I24. Bromide data were collected at the *K* edge, whereas iodide data were collected at 7000 eV, the closest energy to the iodide *K* edge that could be reached. For lysozyme, data for the heavy-metal conditions were collected at the *L*_II_ edge, rather than the more suitable *L*_III_ edge, which has a greater anomalous signal. This less-favourable wavelength was opted for in order to create somewhat poorer conditions for lysozyme, the stable crystals of which are generally known to diffract very well. For proteinase K, data for the heavy-metal conditions were collected at the *L*_III_ edge for all of the conditions except samarium, data for which were collected at the *L*_II_ edge. This is because the *L*_III_ edge was not within the energy range of the beamline. To choose the corresponding energy at the *L*_II_ or *L*_III_ edge, an energy fluorescence scan was performed at the beamline followed by the use of *CHOOCH* (Evans & Pettifer, 2001 ▸).

### 2.4. Data processing   

**Figure 2:**
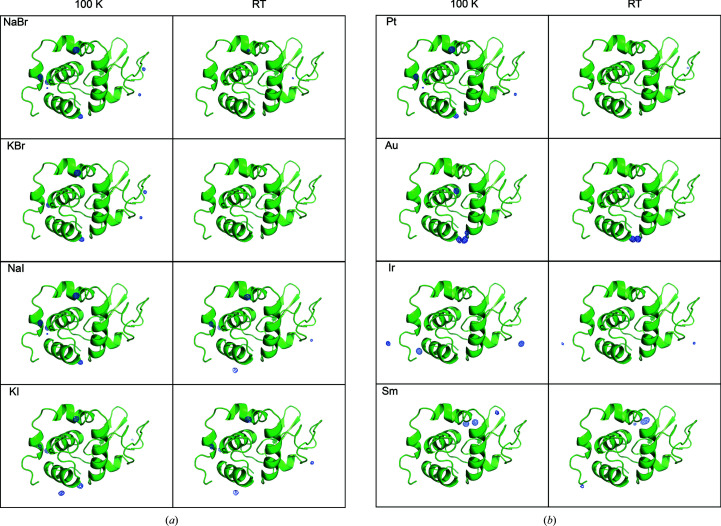
Heavy-atom anomalous signal in lysozyme. (*a*) Halide and (*b*) heavy-metal anomalous signal from diffraction data collected at 100 K (first column) and at RT (second column). The anomalous difference density map (shown in blue) was calculated using *ANODE* (Thorn & Sheldrick, 2011 ▸) and contoured at 10σ. The lysozyme protein backbone is represented as a green cartoon. The σ level was chosen to have strong visible peaks above the noise level. This figure was created using *PyMOL* (DeLano, 2008 ▸).

The data sets were indexed and integrated with *DIALS* (Winter *et al.*, 2018 ▸) and subsequently merged using *BLEND* (Axford *et al.*, 2015 ▸; Foadi *et al.*, 2013 ▸). The resulting merged data sets were scaled with *AIMLESS* (Evans, 2011 ▸; Evans & Murshudov, 2013 ▸); see Supplementary Tables S1, S2, S3 and S4. *HKL*2*MAP* and *SHELXC*/*D*/*E* (Pape & Schneider, 2004 ▸; Sheldrick, 2008 ▸; Usón & Sheldrick, 2018 ▸) were used to phase the derivatives using the SAD and SIRAS methods. The anomalous maps were calculated using *ANODE* (Thorn & Sheldrick, 2011 ▸) for each derivative, as shown in Figs. 2 ▸ and 3 ▸.

## 3. Results and discussion   

The main goal of the present work is to demonstrate experimental phasing and structure solution with data from multiple RT crystals, as described above. In this article, the focus is on *de novo* phasing with a variety of popular heavy-atom derivatives and serial crystal data-collection strategies. Successful experimental phasing was achieved with four derivatives for lysozyme and one derivative for proteinase K.

### 3.1. Phasing at 100 K   

Experimental phasing for both lysozyme and proteinase K was, in general, successful. Details are presented in Tables 3 ▸ and 4 ▸. SAD or SIRAS phasing was attempted using the *SHELX* family of programs (Sheldrick, 2008 ▸; Usón & Sheldrick, 2018 ▸; Pape & Schneider, 2004 ▸). There are three important indicators of success for experimental phasing when using *SHELXC*/*D*/*E*: (i) the cutoff resolution of the average anomalous signal, represented by *d*′′ divided by its standard deviation, (ii) success or failure in the determination of the substructure within 1000 computational trials^1^ and (iii) the fraction of residues fitted into the experimental electron-density map with the autotracing option. With regard to the first indicator, a higher resolution cutoff indicates a stronger anomalous signal derived from more reflections with significant anomalous differences. The third indicator reflects effective phasing because it quantitates the interpretability of an experimental electron-density map; better maps yield a larger number of fitted residues in the automated mode. The three indicators are sequential, interlocked thresholds. Low resolution or failure to find a suitable resolution cutoff as per the first indicator preclude subsequent substructure determination. Similarly, a failure in substructure determination (the second indicator) precludes model building (the fraction of autotracing; the third indicator) because interpretable electron density is not available. There is an obvious difference in phasing effectiveness between lysozyme and proteinase K. Lysozyme is well known to produce high-quality crystals that provide good electron-density maps with nearly all experimental phasing methods. The resolution cutoff in the first indicator for lysozyme and proteinase K averaged 1.70 and 3.32 Å, respectively. Furthermore, anomalous signal for the KBr and iridium derivatives cannot be detected for proteinase K, and the value of the first indicator is very low, 8 Å, for the samarium derivative. Therefore, *SHELXD* cannot find sub­structures for these derivatives. However, phasing and model building are possible in all other cases except for the platinum derivative, where the first indicator has the relatively low value of 4 Å. In addition, the SIRAS methodology within *SHELX* appears to provide better maps than SAD.

### 3.2. Phasing at room temperature   

The results of the phasing experiments for lysozyme and proteinase K data collected at RT are presented in Tables 5 ▸ and 6 ▸, respectively. Compared with cryogenic data, the anomalous signal is markedly lower for data collected at RT. The average value of the first indicator for lysozyme is 2.30 Å (versus 1.70 Å) and that for proteinase K is 4.98 Å (versus 3.32 Å). The lower signal value can be explained by the higher background coming from a popular crystallization plate often used for RT data collection. This means that substructure determination is not possible for a few of the HA derivatives. In general, higher resolutions in the first indicator are associated with substructure determination, but an exception to this rule is found, in the lysozyme case, for the iridium derivative. In contrast, the smaller proteinase K crystals used for RT data collection did not yield as many successes as the crystals at 100 K. Nevertheless, phasing is possible in at least one case: that of the gold derivative. Finally, also at RT SIRAS seems to work better than SAD, still within *SHELX*.

### 3.3. Discussion   

As clearly shown in Tables 3 ▸, 4 ▸, 5 ▸ and 6 ▸, the procedure documents the experimental phasing of crystal structures from serial MX data sets at RT. Not all of the samples or conditions were successful, most obviously because of the large contribution to background noise by the crystallization plates and the weaker diffraction data from the micrometre-sized samples within them. While this use case does not constitute an obstacle for the general application of the methodology, some comments are desirable in order to properly gauge expectations. What can be anticipated from the following in-depth comments is that success or failure are governed by the interplay of several and diverse factors affecting the procedure. These include, but are not limited to, isomorphism of the crystals, the amount of inclusion of HAs in the derivatives, the size of the crystals, degradation owing to radiation damage and the anecdotal and well known intrinsic propensity of certain structures to be experimentally phased with respect to others.

#### 3.3.1. Isomorphism   

The number of crystals used for each derivative and the associated unit-cell variability parameter aLCV (absolute linear cell variation), calculated in *BLEND* and explained in Foadi *et al.* (2013 ▸), are included in Table 7 ▸. The aLCV summarizes the unit-cell size change and its numeric value is closely related to a linear expansion or contraction of the cell, measured in angstroms, with respect to the average unit cell of all crystals considered. This means that a higher aLCV value indicates lower isomorphism. Interesting conclusions from the data collected from RT crystals versus cryocooled crystals can be inferred by simple analysis of the mean and standard deviation of the aLCV for the presented cases (see Fig. 4 ▸). They have a distinct meaning in the present context. The mean aLCV indicates the variability within each of the four combinations: lysozyme at 100 K, lysozyme at RT, proteinase K at 100 K and proteinase K at RT. A higher mean aLCV, *i.e.* non-isomorphism, is more pronounced for proteinase K than for lysozyme both in the 100 K and the RT case. Within each structure, it is also evident that non-isomorphism is higher for cryocooled than for RT crystals; this corroborates the reasons that set us to develop this procedure for RT crystals. The other aLCV statistic, the standard deviation, provides an indication of the unit-cell variability caused by derivatization. Here also we can observe that the HA substrates cause lattice distortion in a greater measure in the cryocooled case. We can also observe that the substrates affect proteinase K crystals more than lysozyme crystals.

#### 3.3.2. The inclusion of HAs in the crystals   

It is clear from the results presented on phasing (Table 5 ▸ and Table 6 ▸) that binding of the heavy atoms to lysozyme occurs at RT in the case of NaI, KI, gold and samarium. For proteinase K, binding can only be ascertained directly from crystallographic data in the case of gold at RT. Figs. 2 ▸ and 3 ▸ display the 10σ contour of the anomalous difference maps, thus calculated to unequivocally show the locations of the peaks. Our results match those reported by Sun *et al.* (2002 ▸), in which short soaking times allow HA ligand binding and are thought to minimize lattice disorder. In the same study, cryocooling was carried out quickly after completing the ligand soak to stop diffusion and lock the heavy atom in the binding position, thereby avoiding lattice degradation.

**Figure 3:**
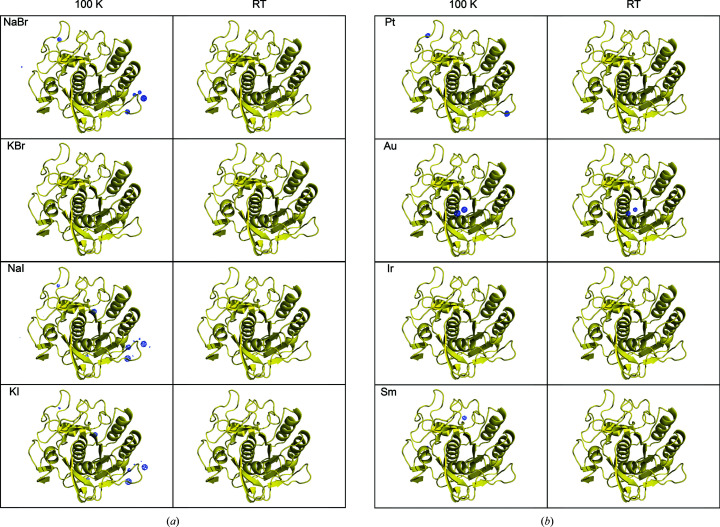
Heavy-atom anomalous signal in proteinase K. (*a*) Halide and (*b*) heavy-metal anomalous signal from diffraction data collected at 100 K (first column) and at RT (second column). The anomalous difference density map (shown in blue) was calculated using *ANODE* (Thorn & Sheldrick, 2011 ▸) and contoured at 10σ. The proteinase K backbone is represented as a yellow cartoon. The σ level was chosen to have strong visible peaks above the noise level. This figure was created using *PyMOL* (DeLano, 2008 ▸).

**Figure 4:**
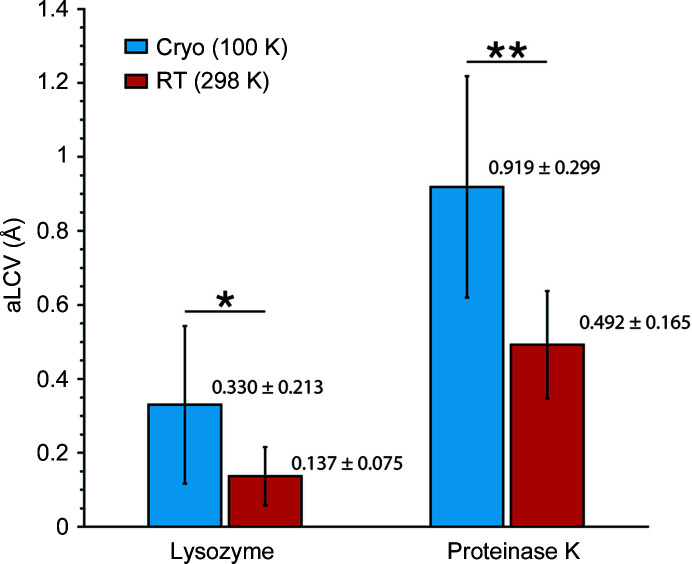
Mean and standard deviation of aLCV for lysozyme and proteinase K at 100 K and RT. Significant differences in aLCV are represented by * (95% confidence) and ** (99%) confidence, as measured by a one-way ANOVA.

**Figure 5:**
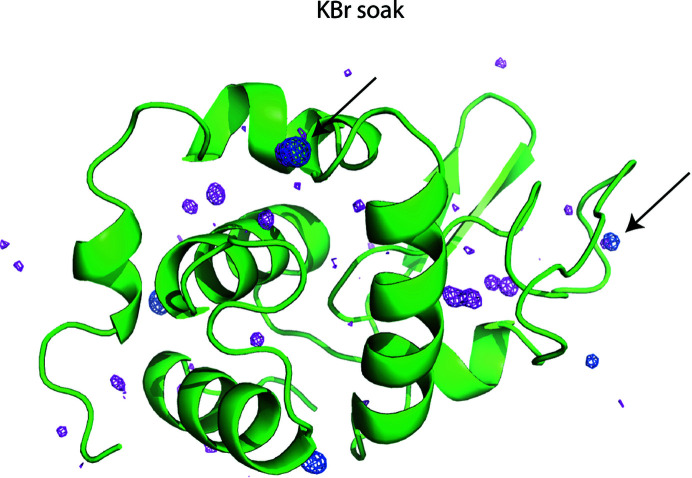
Bromide anomalous signal in lysozyme KBr soaks. Anomalous maps were calculated using *ANODE* (Thorn & Sheldrick, 2011 ▸). The anomalous signal of bromide is displayed at 10σ for diffraction data collected at 100 K (blue) and at 3.5σ for RT data (magenta). The lysozyme backbone is represented as a green cartoon. Black arrows represent anomalous peaks common to the RT and 100 K data sets. The sigma levels were chosen to have strong visible peaks above the noise level. This figure was created using *PyMOL* (DeLano, 2008 ▸).

We compared cryocooled and RT *in situ* HA phasing procedures in this study. The important issue is: have the HAs bound to the structures even if they are not immediately visible in the 10σ anomalous maps from data collected at RT but are present in data collected at 100 K? In our data sets, the anomalous peaks are visible in most of the anomalous maps at 100 K, but are less prominent in RT maps. Anomalous peaks from the all of the HAs are observable in lysozyme RT and/or cryogenic maps (Fig. 2 ▸, Tables 3 ▸ and 5 ▸). This provides clear evidence that heavy atoms do bind to the proteins within the crystal. However, only about half of the HA derivatives produced strong anomalous peaks in RT data sets, whereas all of the cryogenic data did yield *de novo* phasing. The failures to phase are correlated with poor quality of the anomalous signal (see Table 5 ▸). The situation for KBr is illustrative, since this derivative did not yield 10σ anomalous peaks from RT data sets, but does show an anomalous signal at 3.5σ (Fig. 5 ▸). Although the 3.5σ map is noisier, two of the prominent bromide-binding sites identified in the 100 K data sets are also occupied in the RT data sets (see the arrows in Fig. 5 ▸). A similar scenario occurs for all of the HA derivatives of proteinase K, with the exception of KBr and iridium, which both exhibited extremely poor-quality anomalous signal (Fig. 3 ▸, Tables 4 ▸ and 6 ▸). In many cases, the anomalous peaks are visible at the 3.5σ threshold. Derivatization of proteinase K with KBr or iridium appeared to fail since no anomalous peaks could be identified even in the 100 K data sets.

#### 3.3.3. Crystal size   

Proteinase K typically produced smaller crystals than lysozyme (see Section 2.1); the former were about 10 × 10 × 10 µm, whereas the latter were about 200 × 200 × 50 µm. This has obvious repercussions for the diffraction intensities (which are much lower for smaller crystals) and for the lifespan of each crystal during data collection (which is much shorter for smaller crystals) owing to the ensuing radiation damage. Thus, the smaller crystal size for proteinase K meant that the diffraction data were generally of poorer quality than those for lysozyme. An improvement in the quality is obtained when the number of crystals for a data set is substantially higher. Therefore, the number of crystals used for proteinase K was systematically larger than the number of lysozyme crystals. Still, at RT experimental phasing failed for all proteinase K derivatives, with the exception of the gold derivative. It is interesting to note that data from many more crystals (116; see Table 7 ▸) were indeed collected for the gold derivative. This seems to suggest that a larger number of successful cases could have been obtained if data from more crystals had been collected for proteinase K. This observation is also corroborated by the anomalous multiplicity for each derivative at RT for proteinase K (see Table 8 ▸). We can observe that gold indeed has the highest anomalous multiplicity among all derivatives at RT.

#### 3.3.4. Propensity for experimental phasing   

There is ample anecdotal evidence that not all HA ligands enable the same propensity for experimental phasing in the same structure. As summarized in the review by Pike *et al.* (2016 ▸), HA binding depends on multiple factors such as the HA type and charge state, the availability of binding sites in the protein, the crystallization solution and the temperature. One of the major factors is the interaction of the HA with the protein crystallization solution. There is an affinity competition for the HA between the crystallization buffer and the potential binding sites on the protein. The affinity for the latter must be greater for a successful phasing experiment. We assume this to also be the case for the samples analysed in this article. For example, in the lysozyme RT case both iodide substrates led to successful phasing, while this did not happen with the bromide substrates. The iodide substrates also seem to perform better than the bromide substrates in the proteinase K RT case, although no phasing success was obtained. The main reason for including several diverse HA substrates in the procedure was in fact the different propensity for experimental phasing.

## 4. Conclusions   

The procedure for experimental phasing tested and described in this article extends the emphasis on isomorphism for native data described in previous studies (Axford *et al.*, 2015 ▸; Foadi *et al.*, 2013 ▸). Classic derivatization procedures are lengthy and require sustained and substantial manual handling. This affects individual samples in a unique way, thus making the production of isomorphous crystals very difficult. The automated pipeline detailed above reduces human intervention to a minimum, making the production of similar crystals more likely. An important feature characterizing our method is the ready availability of a ‘derivatization kit’ made up of HA compounds which are nontoxic and are regularly used by the community. More hazardous compounds can be added to the kit if the liquid handler is exclusively dedicated to experimental phasing. In fact, the use of a liquid handler for HA soaking is, in such instances, even safer for the operator. For example, in this study, manual sample handling is minimized; the focus is on evaluating derivatization through a systematic analysis of the easy-to-assess indicators described in Section 3.1. To this end, the effect of different HAs on the crystals can be explored using the mean and standard deviation of the aLCV.

The success of this process ultimately depends on the data quality and redundancy. These, in turn, depend on crystal size. Evidently, a higher number of samples are required when the crystals are small, as in the case of proteinase K. However, this is not an insurmountable obstacle, especially in the light of modern serial crystallography at synchrotrons and XFELs.

Serial femtosecond crystallography (SFX) methods at XFEL sources very often exploit RT data-collection strategies using thousands of micrometre-sized crystals, from each of which only one still image is recorded (Chapman, 2019 ▸; Fromme, 2015 ▸; Schlichting, 2015 ▸; Spence, 2017 ▸). The crystals used for SFX are usually similar in size to the proteinase K samples used here. However, very high quality diffraction data from significantly smaller crystals, including submicrometre-sized samples, are often achievable using SFX methods (Gati *et al.*, 2017 ▸; Nass, Redecke *et al.*, 2020 ▸). Consequently, various sample-presentation strategies have been developed to minimize background noise for SFX, because nanometre-sized to micrometre-sized crystals produce weaker diffraction data compared with traditionally sized samples (Awel *et al.*, 2018 ▸; Beyerlein *et al.*, 2017 ▸; Calvey *et al.*, 2019 ▸; Dasgupta *et al.*, 2019 ▸; Davy *et al.*, 2019 ▸; Doak *et al.*, 2018 ▸; Ebrahim, Appleby *et al.*, 2019 ▸; Ebrahim, Moreno-Chicano *et al.*, 2019 ▸; Echelmeier *et al.*, 2019 ▸; Fuller *et al.*, 2017 ▸; Grunbein & Nass Kovacs, 2019 ▸; Lieske *et al.*, 2019 ▸; Martiel *et al.*, 2019 ▸; Meents *et al.*, 2017 ▸; Mehrabi *et al.*, 2019 ▸; Monteiro *et al.*, 2019 ▸; Nass, Gorel *et al.*, 2020 ▸; Nogly *et al.*, 2016 ▸; Oberthuer *et al.*, 2017 ▸; Orville, 2017 ▸; Owen *et al.*, 2017 ▸; Roedig *et al.*, 2017 ▸; Schulz *et al.*, 2018 ▸, 2019 ▸; Shelby *et al.*, 2020 ▸; Sierra *et al.*, 2016 ▸; Stagno *et al.*, 2017 ▸; Suga *et al.*, 2020 ▸; Sugahara *et al.*, 2017 ▸; Weinert *et al.*, 2017 ▸; Wiedorn *et al.*, 2018 ▸; Zhao *et al.*, 2019 ▸). In addition, X-ray pulse durations of tens of femtoseconds and the tight focus and intensity of XFEL pulses often produce very high-quality data sets with little or no radiation-induced effects in the data and refined atomic models. Progress with SFX methods applying *de novo* phasing demonstrates the value of phasing with larger numbers of samples (Gorel, Motomura, Fukuzawa, Doak, Grünbein, Hilpert, Inoue, Kloos, Kovácsová *et al.*, 2017 ▸; Gorel, Motomura, Fukuzawa, Doak, Grünbein, Hilpert, Inoue, Kloos, Nass Kovacs *et al.*, 2017 ▸; Nass *et al.*, 2016 ▸; Yamashita *et al.*, 2017 ▸). However, the extreme limits in XFEL beamtime also highlight the need to develop appropriate methods for *de novo* phasing with serial MX approaches at synchrotrons using smaller samples and potentially RT methods. It is therefore appropriate to extend the method described in this paper to the context of serial crystallography. This will necessitate special attention to the analysis of still images because of the uncertainty in the determination of unit-cell parameters.

## 5. Related literature   

The following reference is cited in the supporting information for this article: Zeldin *et al.* (2013 ▸).

## Supplementary Material

Supplementary Tables containing details of data-collection statistics. DOI: 10.1107/S2059798320009109/qh5065sup1.pdf

## Figures and Tables

**Table 1:** Concentration and soaking time of heavy-metal/halide solutions with 200 nl lysozyme crystal drops for data collected at RT For cryo data collection, the RT soaking time was about 15 min for heavy-metal soaks and 30 min for halide soaks prior to plunge-cooling in liquid N_2_.

HA	Volume ejected (nl)	Stock solution (*M*)	Final concentration (m*M*)	RT soaking time (min)
NaBr	20	2	180.00	10–50
KBr	20	2	180.00	10–25
NaI	20	2	180.00	5–25
KI	20	2	180.00	5–20
K_2_Pt(NO)_4_	20	0.1	9.00	10–50
KAuCl_4_·H_2_O	10	0.1	4.75	5–25
K_2_IrCl_6_	20	0.1	9.00	5–30
SmCl_3_·6H_2_O	20	0.1	9.00	10–25

**Table 2:** Concentration and soaking time of heavy-metal/halide solutions with 200 nl proteinase K crystal drops for data collected at RT For cryo data collection, the soaking time was about 15 min for heavy-metal soaks and 30 min for halide soaks.

HA	Volume ejected (nl)	Stock solution (*M*)	Final concentration (m*M*)	RT soaking time (min)
NaBr	5.0	2	50.00	15–60
KBr	5.0	2	50.00	25–70
NaI	5.0	2	50.00	10–55
KI	5.0	2	50.00	25–70
K_2_Pt(NO)_4_	5.0	0.1	2.50	15–60
KAuCl_4_·H_2_O	2.5	0.1	1.25	5–45
K_2_IrCl_6_	5.0	0.1	2.50	5–40
SmCl_3_·6H_2_O	5.0	0.1	2.50	20–55

**Table 3:** Experimental phasing for lysozyme crystals using data sets collected at 100 K

	200 × 200 × 50 µm lysozyme crystals and data sets collected at 100 K					
	SAD			SIRAS/native cryo		
HA	〈*d*′′/sig〉 anomalous signal resolution cutoff (Å)	*SHELXD* solution	Residues autotraced with *SHELXE* (%)	〈*d*′′/sig〉 anomalous signal resolution cutoff (Å)	*SHELXD* solution	Residues autotraced with *SHELXE* (%)
NaBr	1.50	Yes	98.4	1.50	Yes	85.3
KBr	1.60	Yes	93.0	1.60	Yes	88.4
NaI	2.00	Yes	86.8	2.00	Yes	94.6
KI	1.86	Yes	96.1	1.86	Yes	93.8
K_2_Pt(NO)_4_	1.70	Yes	94.6	1.70	Yes	89.9
KAuCl_4_·H_2_O	1.62	Yes	83.7	1.62	Yes	93.0
K_2_IrCl_6_	1.50	Yes	96.1	1.50	Yes	94.6
SmCl_3_·6H_2_O	1.80	Yes	86.7	1.80	Yes	85.3

**Table 4:** Experimental phasing for proteinase K crystals using data sets collected at 100 K

	10 × 10 × 10 µm proteinase K crystals and data sets collected at 100 K					
	SAD			SIRAS/native cryo		
HA	〈*d*′′/sig〉 anomalous signal resolution cutoff (Å)	*SHELXD* solution	Residues autotraced with *SHELXE* (%)	〈*d*′′/sig〉 anomalous signal resolution cutoff (Å)	*SHELXD* solution	Residues autotraced with *SHELXE* (%)
NaBr	1.90	Yes	98.9	1.90	Yes	97.8
KBr	No signal	N/A	N/A	No signal	N/A	N/A
NaI	2.15	Yes	62.3	2.15	Yes	96.0
KI	2.27	Yes	42.3	2.27	Yes	97.1
K_2_Pt(NO)_4_	4.00	Yes	Failed	4.00	Yes	Failed
KAuCl_4_·H_2_O	1.60	Yes	90.7	1.60	Yes	95.0
K_2_IrCl_6_	No signal	N/A	N/A	No signal	N/A	N/A
SmCl_3_·6H_2_O	8.00	No	N/A	8.00	No	N/A

**Table 5:** Experimental phasing for lysozyme crystals using data sets collected at RT

	200 × 200 × 50 µm lysozyme crystals and *in situ* RT data sets					
	SAD			SIRAS/native *in situ*		
HA	〈*d*′′/sig〉 anomalous signal resolution cutoff (Å)	*SHELXD* solution	Residues autotraced with *SHELXE* (%)	〈*d*′′/sig〉 anomalous signal resolution cutoff (Å)	*SHELXD* solution	Residues autotraced with *SHELXE* (%)
NaBr	2.79	No	N/A	2.79	No	N/A
KBr	2.97	No	N/A	2.97	No	N/A
NaI	2.07	Yes	37.2	2.07	Yes	77.5
KI	2.07	Yes	39.5	2.07	Yes	87.6
K_2_Pt(NO)_4_	2.84	No	N/A	2.84	No	N/A
KAuCl_4_·H_2_O	1.74	Yes	81.4	1.74	Yes	76.0
K_2_IrCl_6_	1.88	No	N/A	1.88	No	N/A
SmCl_3_·6H_2_O	2.07	Yes	63.6	2.07	Yes	79.1

**Table 6:** Experimental phasing for proteinase K crystals using data sets collected at RT

	10 × 10 × 10 µm proteinase K crystals and *in situ* RT data sets					
	SAD			SIRAS/native *in situ*		
HA	〈*d*′′/sig〉 anomalous signal resolution cutoff (Å)	*SHELXD* solution	Residues autotraced with *SHELXE* (%)	〈*d*′′/sig〉 anomalous signal resolution cutoff (Å)	*SHELXD* solution	Residues autotraced with *SHELXE* (%)
NaBr	3.60	No	N/A	3.60	No	N/A
KBr	8.00	No	N/A	8.00	No	N/A
NaI	6.00	Partial	16.1	6.00	No	N/A
KI	3.50	No	N/A	3.50	No	N/A
K_2_Pt(NO)_4_	8.00	No	N/A	8.00	No	N/A
KAuCl_4_·H_2_O	2.60	Yes	9.7	2.60	Yes	95.7
K_2_IrCl_6_	No signal	N/A	N/A	No signal	N/A	N/A
SmCl_3_·6H_2_O	3.15	No	N/A	3.15	No	N/A

**Table 7:** Number of crystals and measure of unit-cell variability for all sets of derivatives

	Lysozyme (200 × 200 × 50 µm)				Proteinase K (10 × 10 × 10 µm)			
	100 K		RT		100 K		RT	
HA	No. of crystals	aLCV† (Å)	No. of crystals	aLCV (Å)	No. of crystals	aLCV (Å)	No. of crystals	aLCV (Å)
NaBr	5	0.14	63	0.29	21	1.07	60	0.71
KBr	5	0.75	25	0.20	19	1.26	55	0.45
NaI	5	0.36	25	0.06	20	0.88	65	0.51
KI	6	0.31	24	0.11	21	0.89	55	0.44
K_2_Pt(NO)_4_	5	0.16	54	0.11	21	0.52	98	0.18
KAuCl_4_·H_2_O	5	0.58	24	0.21	47	1.16	116	0.57
K_2_IrCl_6_	6	0.21	21	0.10	21	0.61	47	0.46
SmCl_3_·6H_2_O	5	0.34	24	0.06	19	0.58	49	0.60

† Absolute linear cell variation calculated in *BLEND* (Foadi *et al.*, 2013 ▸).

**Table 8:** Anomalous multiplicity for crystals of proteinase K

HA	100 K	RT
NaBr	31.9 (12.0)	10.1 (9.6)
KBr	28.0 (10.4)	9.1 (9.0)
NaI	21.8 (7.7)	7.1 (5.5)
KI	24.8 (13.6)	7.8 (7.8)
K_2_Pt(NO)_4_	34.0 (21.0)	11.0 (11.1)
KAuCl_4_·H_2_O	82.9 (66.7)	13.9 (13.9)
K_2_IrCl_6_	31.3 (9.3)	7.5 (7.2)
SmCl_3_·6H_2_O	22.7 (7.4)	7.2 (7.3)

## Footnotes

1 The default in *SHELXD* is 100 trials. In our experience, 1000 trials proved to be a good compromise between efficiency and success. We tried up 10 000 trials for a few cases, but this did not make any difference.
