# Supplementary material for: High-throughput in situ experimental phasing
Source: Acta Crystallogr D Struct Biol. 2020 Jul 28;76(Pt 8):790–801. doi: 10.1107/S2059798320009109 (PMC7397491; doi:10.1107/S2059798320009109)
Supplement: Supplementary file 1 [file d-76-00790-sup1.pdf]

STRUCTURAL BIOLOGY

Volume 76 (2020)

Supporting information for article:

High-throughput *in situ* experimental phasing

Joshua M. Lawrence, Julien Orlans, Gwyndaf Evans, Allen M. Orville, James Foadi and Pierre Aller

Table S1 Data collection and processing statistics for lysozyme at 100 K

Values for the outer shell are given in parentheses.

**Lysozyme**	**Native**	**NaBr**	**KBr**	**NaI**	**KI**	**Pt**	**Au**	**Ir**	**Sm**
Number of datasets	5	5	5	5	6	5	5	6	5
Diffraction source	Synchrotron Diamond Light Source I04								
Wavelength (Å)	0.9795	0.9199	0.9199	1.7712	1.7712	0.8971	0.8994	0.9668	1.6946
Temperature (K)	100	100	100	100	100	100	100	100	100
Beamsize hxv (μm^2)	32x20	32x20	32x20	32x20	32x20	32x20	32x20	32x20	32x20
Detector	Pilatus 6M								
Crystal-detector distance (mm)	289.29	291.5	291.5	169.3	169.3	284.72	323.9	272.2	169.3
Rotation range per image (°)	0.1	0.1	0.1	0.1	0.1	0.1	0.1	0.1	0.1

Total rotation range per crystal (°)	360	360	360	360	360	360	360	360	360
Exposure time per image (s)	0.04	0.04	0.04	0.04	0.04	0.04	0.04	0.04	0.04
Dose per ° rotation (Gy)*	11300	8530	8530	40100	40100	8080	6850	9720	3780
Space group	$P4_32_12$	$P4_32_12$	$P4_32_12$	$P4_32_12$	$P4_32_12$	$P4_32_12$	$P4_32_12$	$P4_32_12$	$P4_32_12$
a, *b*, *c* (Å)	79.05, 79,05, 37.04	78.98, 78.98, 36.89	79.11, 79.11, 36.93	78.94, 78.94, 36.93	78.93, 78.93, 36.91	78.96, 78.96, 37.05	78.49, 78.49, 37.15	78.94, 78.94, 37.08	78.89, 78.89, 36.90
Resolution range (Å)	55.90-1.40 (1.42-1.40)	55.85-1.30 (1.32-1.30)	55.94-1.20 (1.22-1.20)	55.82-1.95 (2.00-1.95)	55.81-1.80 (1.84-1.80)	55.84-1.3 (1.32-1.30)	55.50-1.35 (1.37-1.35)	55.82-1.35 (1.37-1.35)	55.78-1.75 (1.78-1.75)
Total No. of reflections	2304915 (45550)	2578995 (40714)	2412316 (10511)	706829 (30683)	1211201 (12097)	2364181 (39585)	2058536 (32656)	2769469 (61868)	855402 (10350)
No. of unique reflections	23672 (1084)	29268 (1375)	37188 (1772)	8980 (612)	11313 (658)	29423 (1432)	26117 (1267)	26393 (1282)	12271 (654)
Completeness (%)	99.7 (95.1)	99.8 (97.3)	99.8 (98.4)	100 (100)	99.9 (99.4)	99.9 (99.1)	100 (99.7)	100 (100)	100 (99.9)
Anomalous completeness (%)	N/A	99.8 (97.0)	99.3 (88.4)	100 (100)	99.9 (98.3)	99.9 (98.9)	100 (99.7)	100 (100)	99.9 (97.9)
Redundancy	97.4 (42.0)	88.1 (29.6)	64.9 (5.9)	78.7 (50.1)	107.1 (18.4)	80.4 (27.6)	78.8 (25.8)	104.9 (48.3)	69.7 (15.8)

Anomalous redundancy	N/A	46.3 (15.1)	33.7 (3.0)	42.9 (26.1)	57.8 (9.4)	42.3 (14.2)	41.3 (12.9)	55.3 (24.4)	37.3 (8.0)
⟨ $I/\sigma(I)$⟩	47.6 (9.9)	33.7 (7.7)	24.4 (2.1)	34.3 (19.2)	38.8 (8.3)	35.2 (4.3)	17.9 (1.9)	33.9 (8.2)	27.0 (8.4)
$CC_{1/2}$	0.998 (0.993)	1.000 (0.795)	1.000 (0.810)	0.996 (0.993)	0.999 (0.964)	1.00 (0.972)	0.999 (0.905)	0.999 (0.717)	0.995 (0.941)
R_{meas}	0.076 (0.388)	0.164 (1.591)	0.108 (0.603)	0.208 (0.369)	0.151 (0.356)	0.088 (0.743)	0.190 (5.666)	0.153 (1.949)	0.198 (0.680)
$R_{\text{p.i.m.}}$	0.007 (0.057)	0.017 (0.285)	0.012 (0.236)	0.023 (0.050)	0.014 (0.081)	0.009 (0.132)	0.020 (1.062)	0.015 (0.289)	0.023 (0.153)
Overall *B* factor from Wilson plot ($Å^2$)	10.4	7.2	7.8	6.5	8.2	12.4	12.9	10.7	11.0

*Dose calculated with RADDOSE-3D v3.0.794 (Zeldin *et al.*, 2013)

Table S2 Data collection and processing statistics for proteinase K at 100 K

Values for the outer shell are given in parentheses.

**Proteinase K**	**Native**	**NaBr**	**KBr**	**NaI**	**KI**	**Pt**	**Au**	**Ir**	**Sm**
Number of datasets	20	21	19	20	21	21	47	21	19
Diffraction source	Synchrotron Diamond Light Source I24								
Wavelength (Å)	0.9686	0.9191	0.9191	1.7712	1.7712	1.0713	1.0393	1.1047	1.6945
Temperature (K)	100	100	100	100	100	100	100	100	100
Beamsize hxv (μm^2)	9x6	9x6	9x6	9x6	9x6	9x6	9x6	9x6	9x6
Detector	Pilatus 6M								
Crystal-detector distance (mm)	269.2	289.5	289.5	220.1	220.1	232.1	243.0	221.3	220.1
Rotation range per image (°)	0.1	0.1	0.1	0.1	0.1	0.1	0.1	0.1	0.1
Total rotation range per crystal (°)	50	50	50	50	50	50	50	50	50

Exposure time per image (s)	0.01	0.01	0.01	0.01	0.01	0.01	0.01	0.01	0.01
Dose per ° rotation (Gy)*	87400	39600	39600	249200	249200	38000	33400	40600	111000
Space group	$P4_32_12$	$P4_32_12$	$P4_32_12$	$P4_32_12$	$P4_32_12$	$P4_32_12$	$P4_32_12$	$P4_32_12$	$P4_32_12$
a, *b*, *c* (Å)	67.94, 67.94, 102.21	68.08, 68.08, 102.71	68.08, 68.08, 102.50	68.05, 68.05, 102.99	68.09, 68.09, 102.97	67.98, 67.98, 102.45	68.05, 68.05, 102.56	68.07, 68.07, 102.50	68.00, 68.00, 102.51
Resolution range (Å)	56.58-1.40 (1.42-1.40)	56.75-1.30 (1.32-1.30)	56.71-1.30 (1.32-1.30)	56.78-2.10 (2.16-2.10)	56.79-2.20 (2.27-2.20)	56.64-1.40 (1.42-1.40)	56.70-1.40 (1.42-1.40)	56.70-1.30 (1.32-1.30)	56.66-2.00 (2.05-2.00)
Total No. of reflections	2996634 (96135)	3739775 (73035)	3290632 (63902)	614914 (18528)	614841 (29571)	3185673 (98073)	7702308 (319035)	3663010 (59108)	747273 (18739)
No. of unique reflections	47910 (2322)	60093 (2946)	59958 (2917)	14786 (1179)	12919 (1082)	48069 (2342)	48215 (2428)	59945 (3124)	16908 (1195)
Completeness (%)	100 (100)	100 (100)	100 (99.8)	100 (100)	100 (100)	100 (100)	100 (100)	100 (100)	100 (99.4)
Anomalous completeness (%)	N/A	100 (100)	100 (99.7)	100 (99.9)	100 (100)	100 (100)	100 (100)	100 (100)	100 (99.7)
Redundancy	62.5 (41.4)	62.2 (24.8)	54.9 (21.9)	41.6 (15.7)	47.6 (27.3)	66.3 (41.9)	159.7 (131.4)	61.1 (18.9)	44.2 (15.7)
Anomalous redundancy	N/A	31.9 (12.0)	28.0 (10.4)	21.8 (7.7)	24.8 (13.6)	34.0 (21.0)	82.9 (66.7)	31.3 (9.3)	22.7 (7.4)
$\langle I/\sigma(I)\rangle$	9.9 (2.2)	9.4 (1.3)	12.2 (2.5)	17.1 (6.4)	20.4 (9.0)	11.5 (2.3)	13.6 (3.2)	11.6 (1.7)	15.8 (5.9)

$CC_{1/2}$	0.997 (0.750)	0.997 (0.551)	0.998 (0.845)	0.957 (0.955)	0.995 (0.968)	0.998 (0.825)	0.998 (0.916)	0.998 (0.708)	0.992 (0.444)
R_{meas}	0.998 (8.444)	0.619 (6.848)	0.325 (2.140)	0.433 (3.257)	23.251 (497.977)	0.864 (10.725)	2.076 (32.104)	0.340 (2.435)	0.812 (6.337)
$R_{\text{p.i.m.}}$	0.125 (1.307)	0.077 (1.359)	0.043 (0.459)	0.072 (0.823)	3.918 (92.651)	0.105 (1.678)	0.165 (2.822)	0.042 (0.553)	0.129 (1.641)
Overall *B* factor from Wilson plot (Å^2)	5.0	4.8	5.5	7.1	2	6.5	4.8	5.4	7.9

*Dose calculated with RADDOSE-3D v3.0.794 (Zeldin *et al.*, 2013)

Table S3 Data collection and processing statistics for lysozyme at RT *in situ*

Values for the outer shell are given in parentheses.

**Lysozyme**	**Native**	**NaBr**	**KBr**	**NaI**	**KI**	**Pt**	**Au**	**Ir**	**Sm**
Number of datasets	25	63	25	25	24	54	24	21	24
Diffraction source	Synchrotron Diamond Light Source I24								
Wavelength (Å)	0.9686	0.9193	0.9193	1.7712	1.7712	0.9360	0.9027	0.9668	1.6946
Temperature (K)	293	293	293	293	293	293	293	293	293
Beamsize hxv (μm^2)	50x50	50x50	50x50	50x50	50x50	50x50	50x50	50x50	50x50
Detector	Pilatus 6M								
Crystal-detector distance (mm)	269.8	364.2	364.2	220.1	220.1	282.4	296.6	269.9	220.1
Rotation range per image (°)	0.1	0.1	0.1	0.1	0.1	0.1	0.1	0.1	0.1
Total rotation range per crystal (°)	20	20	20	20	20	20	20	20	20

Exposure time per image (s)	0.01	0.01	0.01	0.01	0.01	0.01	0.01	0.01	0.01
Dose per ° rotation (Gy)*	1030	750	750	3640	3640	700	650	1000	3220
Space group	$P4_32_12$	$P4_32_12$	$P4_32_12$	$P4_32_12$	$P4_32_12$	$P4_32_12$	$P4_32_12$	$P4_32_12$	$P4_32_12$
a, *b*, *c* (Å)	79.12, 79.12, 37.95	79.04, 79.04, 38.05	79.09, 79.09, 37.97	79.12, 79.12, 38.11	79.13, 79.13, 38.08	79.11, 79.11, 37.95	78.56, 78.56, 38.26	78.99, 78.99, 38.02	79.10, 79.10, 37.90
Resolution range (Å)	55.95 - 1.25 (1.27-1.25)	55.89 - 1.25 (1.27-1.25)	55.93 - 1.33 (1.35 -1.33)	55.95 - 2.0 (2.05-2.0)	55.95 – 2.0 (2.05-2.0)	55.94-1.27 (1.29-1.27)	55.55-1.33 (1.35-1.33)	55.85 -1.30 (1.32-1.30)	55.93-2.00 (2.05-2.0)
Total No. of reflections	846948 (11016)	2393846 (33423)	891777 (22003)	171271 (2260)	176904 (2442)	2016216 (32455)	815994 (20235)	733057 (14098)	176402 (4706)
No. of unique reflections	33951 (1714)	33977 (1667)	28298 (1385)	8606 (582)	8596 (583)	32403 (1574)	28139 (1374)	30182 (1446)	8542 (583)
Completeness (%)	100 (99.8)	100 (100)	100 (100)	99.8 (97.6)	99.7 (96.5)	100 (100)	100 (100)	99.9 (98.7)	99.6 (97.1)
Anomalous completeness (%)	N/A	100 (100)	100 (100)	97.4 (76.2)	98.0 (79.4)	100 (99.9)	100 (100)	99.8 (96.7)	99.1 (91.9)
Redundancy	24.9 (6.4)	70.5 (20.0)	31.5 (15.9)	19.9 (3.9)	20.6 (4.2)	62.2 (20.6)	29.0 (14.7)	24.3 (9.7)	20.7 (8.1)
Anomalous redundancy	N/A	35.8 (9.8)	15.4 (7.4)	10.0 (2.1)	10.8 (2.3)	31.8 (9.9)	14.6 (7.0)	12.3 (4.8)	10.5 (4.1)
$\langle I/\sigma(I)\rangle$	20.8 (1.8)	20.3 (1.7)	14.0 (1.7)	25.8 (5.1)	21.2 (5.0)	18.6 (1.6)	11.9 (1.6)	19.5 (2.0)	32.7 (11.9)

$CC_{1/2}$	0.999 (0.368)	1.00 (0.615)	0.999 (0.676)	0.996 (0.672)	0.995 (0.662)	0.999 (0.479)	0.998 (0.672)	0.999 (0.469)	0.997 (0.991)
R_{meas}	0.083 (1.831)	0.151 (5.139)	0.148 (2.502)	0.117 (0.586)	0.143 (0.581)	0.144 (3790)	0.174 (3.457)	0.099 (2.471)	0.093 (0.197)
$R_{\text{p.i.m.}}$	0.021 (0.709)	0.023 (1.110)	0.035 (0.613)	0.025 (0.281)	0.029 (0.264)	0.017 (0.818)	0.032 (0.902)	0.019 (0.771)	0.019 (0.065)
Overall *B* factor from Wilson plot (Å^2)	14.1	14.0	14.0	13.7	16.2	13.8	13.0	13.7	17.5

*Dose calculated with RADDOSE-3D v3.0.794 (Zeldin *et al.*, 2013)

Table S4 Data collection and processing statistics for proteinase K at RT *in situ*

Values for the outer shell are given in parentheses.

**Proteinase K**	**Native**	**NaBr**	**KBr**	**NaI**	**KI**	**Pt**	**Au**	**Ir**	**Sm**
Number of datasets	105	60	55	65	55	98	116	47	49
Diffraction source	Synchrotron Diamond Light Source I24								
Wavelength (Å)	0.9686	0.9191	0.9191	1.7712	1.7712	1.0713	1.0393	1.1047	1.6945
Temperature (K)	293	293	293	293	293	293	293	293	293
Beamsize hxv (μm^2)	9x6	9x6	9x6	9x6	9x6	9x6	9x6	9x6	9x6
Detector	Pilatus 6M								
Crystal-detector distance (mm)	290.4	289.5	289.5	220.1	220.1	232.1	243.0	221.3	220.1
Rotation range per image (°)	0.1	0.1	0.1	0.1	0.1	0.1	0.1	0.1	0.1
Total rotation range per crystal (°)	2.5 to 5	5	5	2.5 to 5	5	2.5 to 5	2.5 to 5	5	5

Exposure time per image (s)	0.01	0.01	0.01	0.01	0.01	0.01	0.01	0.01	0.01
Dose per ° rotation (Gy)*	83200	57600	57600	302000	302000	94400	83600	62800	284000
Space group	$P4_32_12$	$P4_32_12$	$P4_32_12$	$P4_32_12$	$P4_32_12$	$P4_32_12$	$P4_32_12$	$P4_32_12$	$P4_32_12$
a, *b*, *c* (Å)	68.46, 68.46, 103.84	68.48, 68.48, 103.90	68.47, 68.47, 103.88	68.29, 68.29, 108.35	68.31, 68.31, 108.12	68.48, 68.48, 103.90	68.50, 68.50, 103.93	68.48, 68.48, 103.90	68.34, 68.34, 108.04
Resolution range (Å)	57.16-1.75 (1.78-1.75)	57.18-1.85 (1.89-1.85)	57.17-2.00 (2.05-2.00)	57.78-2.25 (2.32-2.25)	57.75-2.70 (2.83-2.70)	57.18-1.60 (1.63-1.60)	57.19-1.60 (1.63-1.60)	57.18-1.80 (1.84-1.80)	57.76-2.40 (2.49-2.40)
Total No. of reflections	587973 (16070)	446779 (26289)	323972 (23128)	186097 (13443)	114818 (15207)	760302 (37630)	957768 (47874)	367793 (20724)	152786 (16123)
No. of unique reflections	25659 (1352)	21835 (1319)	17378 (1245)	12791 (1147)	7506 (969)	33411 (1620)	33435 (1632)	23649 (1355)	10608 (1089)
Completeness (%)	100 (100)	100 (100)	100 (100)	100.0 (100)	99.9 (100)	100 (100)	100 (100)	100 (99.9)	100 (100)
Anomalous completeness (%)	N/A	100 (100)	100 (100)	99.9 (99.5)	99.8 (99.9)	100 (100)	100 (100)	100 (99.9)	99.9 (99.8)
Redundancy	22.9 (11.9)	20.5 (19.9)	18.6 (18.6)	14.5 (11.7)	15.3 (15.7)	22.8 (23.2)	28.6 (29.3)	15.6 (15.3)	14.4 (14.8)
Anomalous redundancy	N/A	10.1 (9.6)	9.1 (9.0)	7.1 (5.5)	7.8 (7.8)	11.0 (11.1)	13.9 (13.9)	7.5 (7.2)	7.2 (7.3)
$\langle I/\sigma(I)\rangle$	5.1 (2.0)	3.0 (1.6)	3.1 (2.0)	3.8 (2.0)	4.1 (3.0)	5.0 (2.7)	4.6 (2.3)	3.9 (2.1)	2.8 (1.4)

$CC_{1/2}$	0.923 (0.461)	0.815 (0.503)	0.814 (0.531)	0.905 (0.630)	0.859 (0.643)	0.951 (0.827)	0.954 (0.730)	0.902 (0.616)	0.819 (0.533)
R_{meas}	0.865 (2.816)	1.508 (3.973)	1.477 (3.234)	0.868 (2.708)	0.738 (1.129)	0.843 (3.425)	0.926 (3.825)	1.194 (4.035)	1.148 (2.530)
$R_{\text{p.i.m.}}$	0.176 (0.794)	0.329 (0.880)	0.336 (0.741)	0.224 (0.779)	0.182 (0.278)	0.175 (0.704)	0.171 (0.704)	0.299 (1.024)	0.296 (0.646)
Overall *B* factor from Wilson plot (Å^2)	-2.0	-0.3	-3.6	11.9	-9.0	4.2	4.7	-0.6	4.4

*Dose calculated with RADDOSE-3D v3.0.794 (Zeldin *et al.*, 2013)

Zeldin, O. B., Gerstel, M. & Garman, E. F. (2013). *J. Appl. Cryst.* **46**, 1225-1230.
